# Myconoside and Calceolarioside E Restrain UV-Induced Skin Photoaging by Activating NRF2-Mediated Defense Mechanisms

**DOI:** 10.3390/ijms25042441

**Published:** 2024-02-19

**Authors:** Iva D. Stoykova, Ivanka K. Koycheva, Biser K. Binev, Liliya V. Mihaylova, Maria Y. Benina, Kalina I. Alipieva, Milen I. Georgiev

**Affiliations:** 1 Center of Plant Systems Biology and Biotechnology, 4000 Plovdiv, Bulgarialiliya.vl.mihaylova@gmail.com (L.V.M.);; 2 Laboratory of Metabolomics, Institute of Microbiology, Bulgarian Academy of Sciences, 139 Ruski Blvd., 4000 Plovdiv, Bulgaria; 3 Institute of Organic Chemistry with Centre of Phytochemistry, Bulgarian Academy of Sciences, 1113 Sofia, Bulgaria

**Keywords:** photoaging, keratinocytes, ultraviolet radiation, myconoside, calceolarioside, NRF2

## Abstract

Chronic and excessive ultraviolet (UVA/UVB) irradiation exposure is known as a major contributor to premature skin aging, which leads to excessive reactive oxygen species generation, disturbed extracellular matrix homeostasis, DNA damage, and chronic inflammation. Sunscreen products are the major preventive option against UVR-induced photodamage, mostly counteracting the acute skin effects and only mildly counteracting accelerated aging. Therefore, novel anti-photoaging and photopreventive compounds are a subject of increased scientific interest. Our previous investigations revealed that the endemic plant *Haberlea rhodopensis* Friv. (HRE) activates the antioxidant defense through an NRF2-mediated mechanism in neutrophiles. In the present study, we aimed to investigate the photoprotective potential of HRE and two of its specialized compounds—the phenylethanoid glycosides myconoside (MYC) and calceolarioside E (CAL)—in UVA/UVB-stimulated human keratinocytes in an in vitro model of photoaging. The obtained data demonstrated that the application of HRE, MYC, and CAL significantly reduced intracellular ROS formation in UVR-exposed HaCaT cells. The NRF2/PGC-1α and TGF-1β/Smad/Wnt signaling pathways were pointed out as having a critical role in the observed CAL- and MYC-induced photoprotective effect. Collectively, CAL is worth further evaluation as a potent natural NRF2 activator and a promising photoprotective agent that leads to the prevention of UVA/UVB-induced premature skin aging.

## 1. Introduction

Skin tissue has a complex photo-sensing molecular network as the first line of defense that protects the human body from the harmful effects of environmental stimuli, including ultraviolet radiation (UVR) [1,2,3,4]. Prolonged excessive exposure to solar radiation causes photodamage associated with accelerated skin aging, impairment, oxidative stress, burns, inflammation, erythema, and the breakdown of the extracellular matrix (ECM), as well as visible changes such as wrinkles and dysregulated pigmentation [3,5,6,7,8].

The molecular hallmarks of UVR-induced skin photoaging include excessive reactive oxygen species (ROS) and pro-inflammatory cytokine accumulation along with antioxidant enzyme depletion, lipid peroxidation, decreased collagen synthesis, increased elastin degradation, and hyaluronan deficiency associated with the overexpression of matrix metalloproteinases (MMPs) and dysregulated mitochondrial respiration [3,9,10,11]. Oxidative stress, defined as a disturbance in the balance between ROS production and the antioxidant enzyme system, is involved in the development of many chronic conditions including skin aging [4,5,6,12,13]. Excessive exposure to UVA and UVB rays disrupts the deeper dermal–epidermal junction, which can result in deteriorated skin tissue function that triggers the formation of ROS, leading to DNA damage [4,14,15]. Moreover, it is known that UV-induced ROS overproduction triggers the mitogen-activated protein kinase (MAPK)/activator protein-1 (AP-1) and Janus kinase (JAK)/signal transducer and activator of transcription (STAT) signaling pathways [5,6,7,10,16,17,18]. Concomitantly, keratinocyte self-renewal through Wnt activation induces cell regeneration through non-autonomous signals and subsequent activation of suppressor of the mothers against decapentaplegic homolog (Smad) 3/4 [5,7,19,20]. The transforming growth factor-β (TGF-β)/Smad signaling pathway positively regulates collagen synthesis [5,20,21] along with the proliferation and differentiation of skin tissues, acting in synergy with the Wnt/β-catenin signaling pathway. Excessive UV exposure inhibits type I procollagen synthesis through the downregulation of the type II receptor of TGF-β1 and the upregulation of Smad 7 [7,20,21]. Precisely, the maintenance of skin homeostasis in the context of the equilibrium between UV-triggered MMP expression and the biosynthesis of collagen is a key part of the UV-induced mechanisms of ECM remodelling associated with visible signs of photoaging, e.g., wrinkles, epidermal thickening, and dysregulated skin tone [1,4,5,7,21,22]. In this sense, damage to the collagen layer in photoaged skin is linked to increased intracellular ROS accumulation [4,5,14].

The redox-sensing transcription factor nuclear factor erythroid 2-related factor 2 (NRF2) has recently emerged as a potential therapeutic target in skin cells, like epidermal keratinocytes and dermal fibroblasts, and as a major player in photoaging development [4,15,22,23,24,25]. The pharmacological modulation of NRF2 is exploited in the context of numerous human pathologies such as non-communicable chronic diseases (NCDs) or oxidative stress-related pathologies [4,25,26,27]. Exposure to ionizing radiation and solar UVR induces ROS-mediated oxidative stress in the upper epidermal skin layers that expose the NRF2 signaling pathway as critical for photoaging development [4,23,24,28,29,30,31]. As a result of excessive ROS accumulation in skin cells, NRF2 dissociates from the complex with its negative regulator Kelch-associated protein 1 (KEAP1) and translocate into the nucleus. Activated NRF2 binds to the antioxidant response element (ARE)-containing genes to regulate various antioxidant enzymes, including superoxide dismutase (SOD), heme oxygenase 1 (HO-1), and NAD(P)H quinone oxidoreductase 1 (NQO1), participating in cellular redox homeostasis regulation and protecting the skin against the destructive effects of ROS [23,24,25,26,27,28,29,30,31]. In parallel with NRF2, the peroxisome proliferator-activated receptor-gamma coactivator (PGC-1α) regulates the generation of mitochondrial ROS, which exacerbates oxidative skin photodamage [4,24,32,33,34]. Following UVB irradiation, mitochondria-generated H_2_O_2_ can circulate freely in the cytosol, further increasing cellular free radical levels and causing the accumulation of oxidative stress [2,4,5,22,23,24,25,26,27,28,29,30,31,32,33,34].

The development of effective sunscreen agents with environmental soundness remains a challenge for the pharmaceutical and cosmetic industries, despite growing knowledge of the mechanisms of photoaging [35,36,37,38]. Currently, the application of sunscreen products (with different UVA, UVB, or both UVA/UVB absorption ranges and sun protection factors) is the main skin photoprotective option against the harmful effects of UV light apart from limitation of direct sunlight exposure time [2,6,7,10,35,36,37,38]. The photoprotective active ingredients approved for use in sunscreen products within the United States of America (by the Food and Drug Administration agency) and the European Union are restricted to a limited number of compounds due to data concerning their long-term toxicity and adverse effects [2,35]. The available sunscreens such as avobenzone, benzophenone-3 (oxybenzone), octisalate, octocrylene, homosalate, para-aminobenzoic acid (PABA), octinoxate, titanium dioxide, and zinc oxide protect the skin against photodamage, but they have some adverse effects, such as irritation and dryness, as well as weak photostability [2,5,6,35,36,37,38,39]. For example, the most commonly utilized benzophenone-3 has been reported to promote skin irritation, oxidative stress, and cell death, as well as allergic reactions [7]. Similarly, titanium dioxide and zinc oxide have been described in the context of their adverse effects on the skin, such as excessive oxidative stress generation and allergies [7,37]. Several major concerns exist about UV filters’ toxicity in long-term use such as their possible absorption through the skin, the risk of apoptosis induction or elevated mutagenesis. In addition, insufficient knowledge of interactions between sunscreens and the skin and also the negative impact of sunscreens on the environment (such as the red coral reefs), fuel the debate for restriction of their use [2,7,35,36,37]. Specialized plant secondary metabolites such as rutin, ferulic acid, caffeic acid, caffeine, and quercetin synthesized in response to solar simulation could serve as natural photoprotective substances with high photostability and low phototoxicity that absorb UVA/UVB radiation, decrease ROS formation, and could improve the efficacy of available UV filters [1,10,14,22,28,37,38,39,40,41,42,43]. For instance, avobenzone-based [38] or PABA-containing [39] topical formulations that include rutin have been reported, with increased photostability of the active UV filter and improved photoprotective effects [38,39,40]. Similarly, improved clinical efficacy in regards to increased SFP has been reported for ethylhexyl triazone- and bis-ethylhexyloxyphenol methoxyphenyl triazine-containing topical formulations upon the addition of ferulic acid [40,41]. Caffeine combination with different UV filters, namely, ethylhexyl methoxycinnamate, avobenzone, and titanium dioxide, induced improved SPF values in both in vitro and in vivo assays [42]. Furthermore, a number of studies have pointed out the potential of plant-derived compounds such as resveratrol, kaempferol, quercetin, and gallic acid to replace the available UV filters [18,20,22,40,43].

The resurrection plant *Haberlea rhodopensis* Friv. (family Gesneriaceae), known as the Orpheus flower, is an endemic species in the Balkans [44,45,46]. Several decades of research have been dedicated to the investigation of the phytochemical composition, unique desiccation tolerance, and therapeutic potential of this plant [27,44,45,46]. The traditional use of *H. rhodopensis* is based on its tonic, immunomodulating, anti-inflammatory, and wound-healing properties [38,39,40]. A previous study of ours identified two biologically active phenylethanoid glycosides, namely calceolarioside E (CAL) and myconoside (MYC), isolated from the in vitro propagated *H. rhodopensis* [28]. Both compounds regulate the senescence-associated markers in neutrophils and cellular redox homeostasis through an NRF2-mediated mechanism [28]. However, their ability to protect skin cells from the harmful influence of UV radiation is not clarified.

In the present study, we investigated the potential photoprotective effect of *H. rhodopensis* extract (HRE) and its constituents CAL and MYC in an in vitro photoaging model in human epidermal keratinocytes exposed to UVA/UVB irradiation. Furthermore, the underlying signaling molecular pathways involved were explored, with special emphasis on the cellular redox defense mechanisms.

## 2. Results

### 2.1. Pre-Treatment with H. rhodopensis Extract and Its Secondary Metabolites Induces Photoprotective Effects in UVA/UVB-Exposed Human Keratinocytes

**Figure 1:**
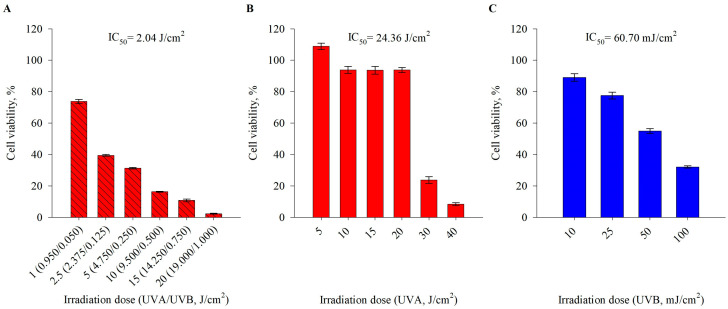
Phototoxicity irradiation doses of UVA/UVB (**A**), UVA (**B**), and UVB (**C**) on cell viability in human keratinocytes. For each irradiation mode, the half-maximal inhibitory concentration (IC_50_) was calculated. Error bars indicate the mean ± SEM for cell viability expressed as a percentage from the native dark control.

To select the biologically effective dose of UVR to stimulate HaCaT cells, we performed a viability assay to acquire the half-maximal inhibitory concentration (IC_50_) of UVA, UVB, and the combination of UVA/UVB (Figure 1). Despite the fact that human dermal fibroblasts are more commonly utilized in photoaging research, the HaCaT keratinocyte cell culture is a useful in vitro model to investigate UVR-induced phototoxicity effects [12,15,18,22,29]. Cell viability was remarkably affected by UVA radiation at irradiation doses higher than 30 J/cm^2^ (Figure 1B), while UVB-induced phototoxicity resulted in a dose-dependent decrease in cell viability up to 32.0% and 2.3%, respectively, at UVB doses over 50 mJ/cm^2^ (Figure 1C). The combined UVA/UVB irradiation was set at a ratio of 95:5% to mimic sunlight-induced photodamage. An exponential dose-dependent increase in phototoxicity was observed following exposure to UVA/UVB stimulation over 2.5 J/cm^2^ in human keratinocytes, which was reached upon 30 min of irradiation. Based on the calculated IC_50_ values for UVA/UVB (ratio 95:5%), we selected the irradiation dose of 2.5 J/cm^2^ for the further experimental workflow. The respective time that was required to reach the set photon energy dose was determined by the technical specifications of the irradiation chamber, and for this irradiation dose, it was 30 min (Figure 1A).

**Figure 2:**
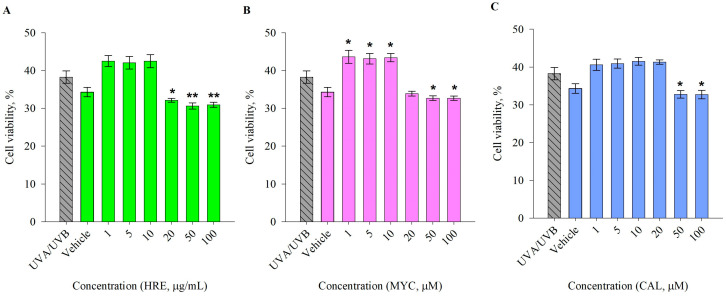
Photoprotective effects of *Haberlea rhodopensis* extract (HRE; (**A**)), myconoside (MYC; (**B**)), and calceolarioside E (CAL; (**C**)) in UVA/UVB-exposed human keratinocytes. Error bars indicate the mean ± SEM for cell viability expressed as a percentage from the dark control. Statistical significance between the groups was determined via one-way ANOVA, followed by Tukey’s post hoc test; * *p* < 0.05 and ** *p* < 0.01 compared to the UVA/UVB group.

Next, with the selected exposure parameters of UVA/UVB 2.5 J/cm^2^ for 30 min, we evaluated the eventual phototoxicity of pre-treatment with HRE, as well as that of pure MYC and CAL (Figure 2). The treatments were applied 1 h prior to UVA/UVB irradiation and left for 24 h. They all demonstrated a lack of phototoxic effects at concentrations up to 10 μg/mL for HRE and 10 and 20 μM for MYC and CAL, respectively. However, solely MYC application provided significant elevation in cell viability following UV-induced damage. Therefore, we applied HRE in treatment concentrations of 1, 5, and 10 μg/mL for the subsequent biological analyses, while the pure compounds MYC and CAL were added at 1, 5, or 10 μM.

### 2.2. Myconoside and Calceolarioside E Alleviate UVA/UVB-Induced ROS Generation in Human Keratinocytes

**Figure 3:**
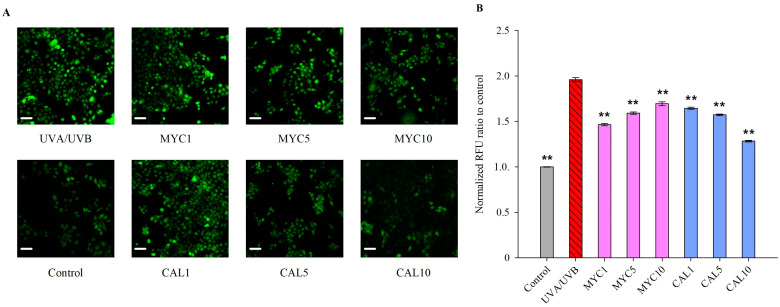
Myconoside (MYC) and calceolarioside E (CAL) reduce UVA/UVB-induced ROS production in human keratinocytes. The fluorescence images of the experimental groups stained with DCF-DA reagent after UVA/UVB irradiation were observed at 20x magnification (scale bar = 50 μm) with an FITC filter (**A**). Quantification of the normalized fluorescence intensity of intracellular ROS generation in HaCaT cells (**B**). The quantification of the stained oxygen radicals was measured as an average pixel intensity using ImageJ software version 1.53t and was represented as the normalized pixel intensity against the UVA/UVB group. Statistical significance between the groups was determined via one-way ANOVA, followed by Tukey’s post hoc test; ** *p* < 0.01 compared to the UVA/UVB group.

Intracellular ROS accumulation was determined using a DCF-DA fluorescent probe [47]. UVA/UVB exposure resulted in a remarkable increase in intracellular ROS production in human keratinocytes (Figure 3). Pre-treatment with MYC significantly reduced these ROS levels at all treatment concentrations. Similarly, CAL reduced the accumulated oxygen species at all experimental concentrations and in a dose-dependent manner. The highest concentration of 10 μM applied of CAL reduced the intracellular ROS to levels comparable to that of the non-irradiated dark control keratinocytes.

Collectively, the observed reduction in UV-induced ROS formation suggested the ability of both MYC and CAL to inhibit the oxidative stress characterizing the process of photoaging.

### 2.3. Myconoside and Calceolarioside E from H. rhodopensis Modulate the Gene Expression Profile of UVA/UVB-Irradiated Keratinocytes

**Figure 4:**
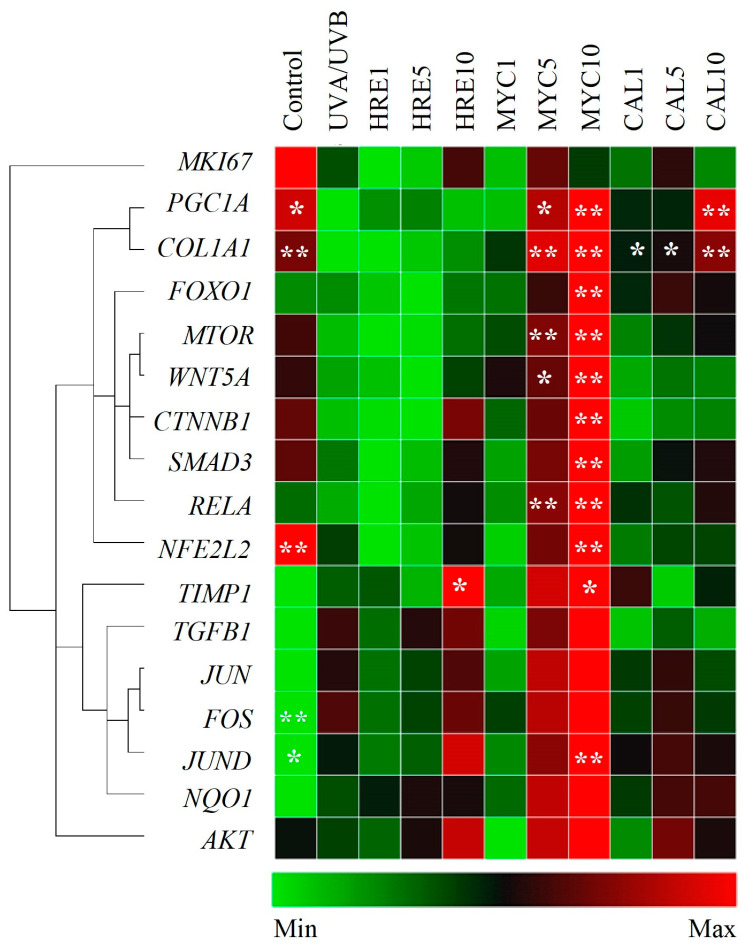
Gene expression profile modulation associated with UVA/UVB-induced photoaging by *H. rhodopensis* extract (HRE), calceolarioside E (CAL), and myconoside (MYC). Clustergram and heatmap of the relative gene expression analysis from the RT-qPCR. The results are expressed as the mean ± SEM compared to the UVA/UVB-exposed model group from three independent experiments. * *p* < 0.05 and ** *p* < 0.01 compared to the photoaging model group.

As a result of increased oxidative stress and inflammation, excessive UVR exposure decreases collagen content within the ECM and contributes to dysregulated cell death mechanisms in both keratinocytes and dermal fibroblasts [9,10,11,12]. The gene expression analysis showed that the collagen type I encoding gene (*COL1A1*) mRNA levels were increased upon pre-treatment with MYC and CAL compared to the UVA/UVB-irradiated controls (Figure 4). The downregulation in *COL1A1* expression that resulted from UV exposure [18,20,21,22] was associated with a simultaneous decrease in the expression of *TGFB1*, *SMAD*, and *TIMP* (Figure 4). TGF-1β regulates the production of procollagen and is involved in matrix collagen synthesis via the TGF-1β/Smad signaling pathway [7,12,20,47,48,49]. Among the selected treatments, solely MYC upregulated the mRNA expression of *TGFB1* and *SMAD3*. Intriguingly, the *MMP1* and *CASP3* levels were downregulated in the UVR-exposed cells in comparison with the controls [49,50], which could be due to compensatory mechanisms or negative feedback loop upregulation resulting from their initial depletion (Supplementary Figure S3).

The Wnt/β-catennin signaling governs cell proliferation, migration, and cellular regeneration following UVR-induced skin damage [7,8,10]. The gene expression data indicated that MYC, but not HRE or CAL, positively regulated the mRNA expression of *WNT5a* and *CTNNB1*. In addition, genes from PI3K/AKT/mTOR/FOXO signaling were detected as being critical for the regulation of UV-stimulated dysregulated keratinocyte proliferation [3,6,31,51,52,53]. Activation of *MTOR* and modulation of its downstream target *FOXO1* was observed upon pre-treatment with MYC at a 10 μM concentration compared to UVR-stimulated keratinocytes.

Consistent with previous reports [50,54,55], upregulation of genes related to the MAPK/AP-1 signaling pathway (*c-JUN*, *JUND,* and *c-FOS*) was observed via UVA/UVB irradiation, even though not with a sufficient level of significance. Interestingly, the commonly known skin aging marker genes such as *MMP1* were found to be negatively regulated upon UVR stimulation.

Reduced PGC-1α (encoded by the *PPARGC1A* gene) is associated with disturbed NAD^+^ homeostasis, mitochondria dysfunction, and accelerated skin aging [33,34]. Properly functioning NRF2 is of critical importance for cellular redox balance, hence the defense against UV-induced photodamage in keratinocytes [4,29,30,31,32]. The detected elevated levels of intracellular ROS (Figure 3) could be correlated with the observed downregulation of the mRNA levels of *PPARGC1A* and *NFE2L2* detected within the UV-exposed model group [32,33,34]. Both MYC and CAL pre-treatment resulted in significant upregulation in *PPARGC1A* expression while *NFE2L2* was only restored upon MYC application (Figure 4).

Collectively, the data from the gene expression analysis suggest that both MYC and CAL modulate signaling pathways related to the UVR-induced photodamage response.

### 2.4. Calceolarioside E Activates the Transcription Factor NRF2 in UVR-Stimulated Keratinocytes

**Figure 5:**
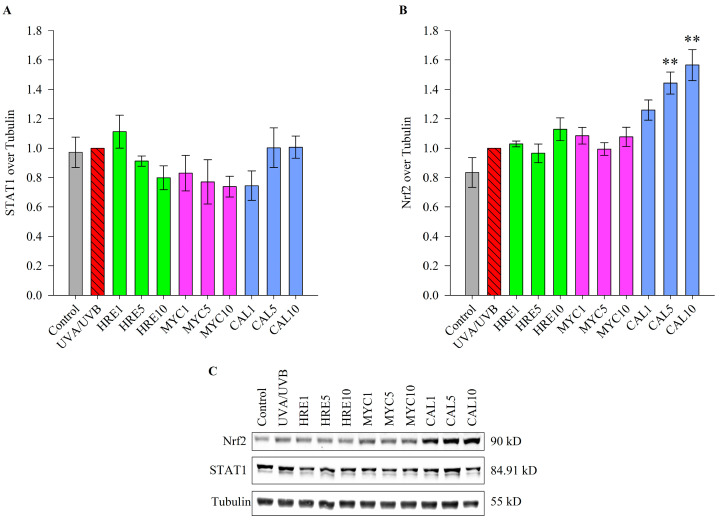
Calceolarioside E (CAL) activates NRF2 signaling in UVA/UVB-photoaged HaCaT cells. Western blot of STAT1 (**A**) and NRF2 (**B**) 24 h after UVA/UVB irradiation and 1 hr pre-treatment with HRE (1, 5, and 10 μg/mL), MYC, or CAL (1, 5, and 10 μM) and representative bands from the Western blot analysis (**C**). The results are presented as the mean ± SEM from three independent experiments. ** *p* < 0.01 compared to the UVA/UVB group.

To further clarify the molecular mechanism of the photoprotective effect of HRE and pure MYC and CAL in UVR-stimulated keratinocytes, we detected the protein levels of STAT1 as a major transcription factor involved in the skin inflammatory response and autophagy [7,16,56] and the protein levels of NRF2 as the master regulator of the oxidative stress response [4,23,24,29,30,31,32]. The Western blot data showed that the abundance of STAT1 protein was not affected upon treatment with either HRE or any of its pure compounds in UV-irradiated keratinocytes (Figure 5A).

In the cytoplasm, the NRF2 transcription factor is bonded in a complex with its negative regulator KEAP1 [23,24,29,30,31,32]. However, disruption in cellular redox homeostasis leads to the sequestration of KEAP1 and an increase in the free active form of NRF2 and its nuclear translocation. In this study, we observed that pre-treatment with CAL (1–10  μM for 1 h) dose-dependently increased the total NRF2 protein levels in UVR-exposed human keratinocytes (Figure 5B). This was also corroborated by a proportional decrease in UV-induced intracellular ROS formation upon CAL treatment (Figure 3).

The protein data clearly inferred that CAL favors the activation of Nrf2 to mediate its antioxidative downstream effects.

### 2.5. Proposed Model Mechanism of the Anti-Photoaging Activity of MYC and CAL Isolated from H. rhodopensis Extract

**Figure 6:**
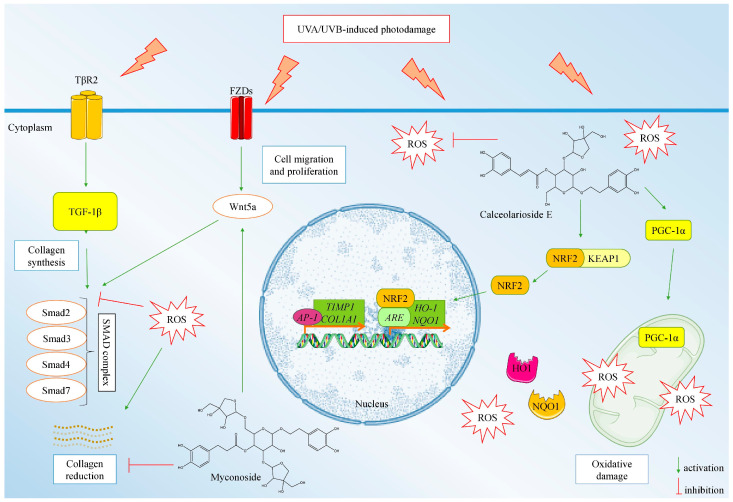
Molecular-based modeling of the anti-photoaging activity of myconoside (MYC) and calceolarioside E (CAL) mediated through NRF2/PGC-1α and TGF-β/Smad/Wnt signaling in UVA/UVB-exposed HaCaT cells.

The integrated data from the ROS formation assay and the gene and protein expression analyses suggest that the observed photoprotective potential of HRE is mainly mediated through its pure compounds MYC and CAL. We have proposed a model mechanism of action of the anti-photoaging activity of both MYC and CAL (Figure 6).

In regards to the pre-treatment with MYC, at its highest experimental concentration, it upregulated the mRNA expression of multiple players within the AKT/mTOR/FOXO, PGC-1α/NRF2, and TGF-β/Smad/Wnt signaling pathways. These observations demonstrate that MYC counteracts ROS formation through PGC-1α activation and stimulates collagen synthesis through a TGF-β/Smad/Wnt-mediated mechanism. Calceolarioside E pre-treatment upregulated in a concentration-dependent manner the PGC-1α encoding gene and *COL1A1,* which is in correlation with the detected dose-dependent NRF2 activation and the reduction in intracellular ROS levels. Therefore, we could speculate that CAL protects HaCaT keratinocytes against solar UV-induced photodamage through NRF2-mediated activation of cellular redox defense mechanisms.

## 3. Discussion

Photoaging is characterized by a loss of skin elasticity with the depletion of collagen, elastin, and hyaluronan content, resulting in coarse wrinkling and dysregulated pigmentation [1,2,3,4,5,6,7,8]. In the present study, UVA/UVB irradiation was used to mimic the exposure close to the solar spectrum that is mostly associated with skin photoaging induction. Decreased cell viability due to UVR-induced ROS generation and inflammation are among the first molecular signs of premature skin aging [4,22,23,24,25]. Our findings demonstrated that UVA/UVB exposure reduced cell viability, induced ROS overproduction, and disturbed genes from the TGF-β/Smad/Wnt-mediated collagen synthesis in HaCaT cells.

The resurrection plant *H. rhodopensis* has long been an object of intense research interest as it holds promise to reveal unique molecular mechanisms of drought tolerance that could be transferred to other plant species. Its extreme resistance to harsh environmental conditions has been reflected within its specific phytochemical profile of specialized/secondary metabolites [38,40]. Several studies have reported that *H. rhodopensis*’ anti-inflammatory and immunomodulating potential could be attributed to specific secondary metabolites [44,45,46]. Among the secondary metabolites of HRE, the phenylethanoid glycosides MYC and CAL are the most commonly associated with the beneficial biological activity of the plant extract [28,46]. However, the molecular mechanism involved in the beneficial effect of MYC or CAL treatments is largely unknown. An earlier study proposed that topical application of HRE improves human skin elasticity and increases collagen levels, which has been attributed to the high content of MYC [39]. Our previous data revealed the potent NRF2 activating potential of CAL isolated from *H. rhodopensis* in murine neutrophils [28]. Several studies have reported that MYC decreases ROS in the state of increased oxidative stress in various tissues, attenuates inflammation, and modulates cellular senescence [28,46]. The present study demonstrates and confirms the potential of the photoprotective mechanisms of *Haberlea* extract and its secondary metabolites myconoside and calceolarioside E.

Increased oxidative stress induced by UVR exposure is normally controlled by the endogenous natural antioxidant system governed mainly by the KEAP1/NRF2 signaling pathway [4,15,22,23,24,29,30,31,32]. UV-induced modification of lipid peroxidation and ROS production is associated with increased NQO1, SOD, and HO-1, which are all controlled by the NRF2 transcription factor. The pharmacological modulation of NRF2 as a novel molecular target has been explored in the context of skin photoprotection [4,22,23,24,26,27,28,29,30,31,32]. A solid number of studies have reported the potential of plant-derived bioactive leads to modulate the redox-sensitive transcription factor NRF2, which was recently reviewed in detail by Chaiprasongsuk and Panich [43]. For instance, tanshinones isolated from *Salvia miltiorrhiza* have been identified as potent natural NRF2 inducers that reduce UV-mediated skin photodamage [24]. Similarly, the natural sesquiterpene zerumbone diminished UVA-induced photodamage and premature skin aging through NRF2/ARE-mediated mechanisms [47]. The present study demonstrates that UVR stimulation increases oxidative stress levels, and both MYC and CAL acted as natural antioxidants as they reduced the ROS content. Furthermore, CAL treatment increased the protein expression of NRF2 in HaCaT cells with induced photoaging, which is in agreement with our previous report on murine neutrophils [28].

Mitochondrial respiration and cellular redox homeostasis are coordinated through a PGC-1α/NRF2 interrelated mechanism. Moreover, in keratinocytes, PGC-1α acts as a key regulator of terminal differentiation and DNA damage response [33,34,57,58]. Within the present study, both MYC and CAL positively regulate PGC-1α expression, which could be associated with the observed reduction in ROS levels through increased mitochondrial biogenesis.

Oxidative stress and inflammation have a close interaction that leads to the progression of photoaging under UVR exposure. Oxidative stress is involved in collagen degradation in the skin, and inflammation stimulates epidermal thickening and reduces collagen contents in the epidermis and dermis [1,5,7,22,59,60,61,62]. The MAPK family of kinases are major regulators involved in cell apoptosis and inflammatory responses that trigger the phosphorylation of c-Fos, c-Jun, and JunD proteins (AP-1 complex), leading to collagen degradation via upregulation of MMP-1, -3, and -9 [1,5,6,7,54,55,59,60,61,62]. Activation of specific MAPK inhibitors, such as JNK and P38/MAPK, by blocking the Smad 3/4 complex has been suggested to suppress TGF-β expression, thereby impairing collagen synthesis [5,11,59,60,61,62]. Additionally, the JAK/STAT signaling pathway plays a critical role in skin UV-induced inflammation and autophagy during the photoaging process [16,17]. Also, over-activation of PI3K/AKT/mTOR in UV-mediated photodamage related to skin tissue homeostasis and function through mitophagy regulation [13,31,53] and cell proliferation and survival [30,54]. Furthermore, the UV-promoted PI3K/AKT signaling cascade plays a role in mediating ROS, triggering certain cellular events, such as proliferation, differentiation, and inflammation, and the AKT-mediated regulation of MAPKs is a strategy to protect against UV-induced skin damage and inflammation [54]. Apart from its role in the cleavage of pro-apoptotic substrates, caspase-3 has been proposed to regulate cell cycle progression in epidermal cells as it cleaves α-catenin and hence promotes the release of yes-associated protein (YAP) from an α-catenin/YAP complex and stimulates a proliferative response [48]. The transcriptional data exposed MYC as the most active within the used experimental treatments in UVR-stimulated human keratinocytes as it upregulated multiple genes from the TGF-β/Smad/Wnt, P3K/AKT/mTOR, and PGC-1α/NRF2 signaling pathways.

Excessive UV exposure inhibits type I procollagen synthesis by downregulating the type II receptor of TGF-β1 and upregulating Smad7 levels. Epidermal monolayer repair is stimulated by increasing collagen turnover co-ordinately with TGF-β1 expression [7,12,20,50]. Anti-photoaging agents should stimulate the TGF-β1/Smad pathway in the skin after UV radiation. TGF-β1 regulates the production of procollagen and is involved in matrix collagen synthesis via the TGF-β1/Smad signaling pathway. Wnt signaling is involved in cell proliferation and migration, and it is associated with cellular regeneration after UV radiation [11,12,20]. Wnt signaling and TGF-β1 interact with each other, and Wnt signaling is required for TGF-β signaling in fibrosis [11,19,20]. The present study demonstrated that UVR exposure induced cell death by increasing oxidative stress and suppressing TGF-β1/Smad/Wnt signaling in HaCaT cells, which could be prevented through pre-treatment with HRE-derived MYC and CAL. Our hypothesis is that the activation of TGF-β/Smad3 and the non-canonical Wnt pathway upon MYC pre-treatment is related to *COL1A1* synthesis and the regeneration of cell–cell communication. Notably, HRE extract has no effects on the expression of collagen and MMP1. However, HRE extract pre-treatments and that with pure MYC and CAL ameliorated the UVA/UVB-induced decrease in cell viability.

Due to their natural origin and promising bioactivity, these small molecules might be included in sunscreens in alcohol-based or lipophilic formulations for application in topical products for delivery into the *stratum corneum* to protect the skin from UV radiation [37,38,39,40,41,42,43] as an alternative or adjunct to the available chemical UV-filters. Further experiments on 3D skin reconstruction models or in vivo animal models of photodamage have to explore how their possible incorporation into topical formulations interacts with the human skin, to follow up on whether or how deep they penetrate into skin layers, to track the effects of different carriers on MYC and CAL stability as well as for potential side effects.

Collectively, our findings provide insights into the anti-photoaging activity of in vitro propagated *H. rhodopensis* extract and pure myconoside and calceolarioside E in UVR-stimulated human keratinocytes. Mechanistically, both MYC and CAL exert the potential to modulate the synthesis of collagen and activate photoprotective mechanisms in UVA/UVB irradiation through modulation of redox homeostasis and decreased ROS accumulation. Calceolarioside E acts as a potent NRF2 activator while MYC stimulates PGC-1α and TGF-1β/SMAD/Wnt signaling pathways. These data provide a rationale for the further development of topical products based on the biologically active compounds from *H. rhodopensis* extract against UV-induced premature skin aging.

## 4. Materials and Methods

### 4.1. Materials

Dulbecco’s modified Eagle medium (DMEM) with high glucose 4.5 g/L (#D5796), fetal bovine serum (#F7524), penicillin/streptomycin/amphotericin B (#A5955), trypsin-EDTA (#59418C), 3-(4,5-dimethylthiazol-2-yl)-2,5-diphenyltetrazolium bromide (MTT); RNAzol RT reagent (#R4533), Bradford reagent (#B6916), RIPA lysis buffer (#R0278), and protease and phosphatase inhibitor cocktail (#PPC1010) were obtained from Merck KGaA (Darmstadt, Germany). Buffers and chemicals used to perform electrophoresis, Western blot analysis, and quantitative real-time polymerase chain reaction (RT-qPCR) were acquired from Bio-Rad Laboratories Inc. (Hercules, CA, USA). The following primary antibodies were used: rabbit anti-STAT1 (#14994), anti-NRF2 (#12721) from Cell Signalling Technology (Leiden, The Netherlands) and rabbit anti-tubulin (#12004166) and goat anti-rabbit IgG StarBright Blue 700 (#12004162) antibodies from Bio-Rad. All other materials and analytical-grade substances were delivered from Merck KGaA (Darmstadt, Germany) unless otherwise specified.

Myconoside (molecular weight 744.7 g/M) and calceolarioside E (molecular weight 478.4 g/M) were isolated from crude methanol extract of in vitro cultivated *H. rhodopensis* aerial parts according to the protocol described by Amirova et al. [28].

### 4.2. Cell Culture, Irradiation, and Treatment

The spontaneously immortalized human epidermal keratinocyte (HaCaT; Cell Line Service GmbH, Eppelheim, Germany) cells were cultured according to the previously described conditions [56].

The multi-channel UV irradiation chamber BS-02 by Opsytec Dr. Gröbel GmbH (Ettlingen, Germany) equipped with 4 light UVB sources (Sankyo Denki, Japan; UV light intensity—5 mW/cm^2^; an emission spectrum between 208 and 315 nm; λ_max_ = 306 nm), 4 UVA sources (Sankyo Denki, Japan; UV light intensity—8 mW/cm^2^; an emission spectrum between 315 and 400 nm; λ_max_ = 352 nm), an external UV-MAT dose-controller (#820920), and an area attenuator was used to irradiate the HaCaT cells. Non-irradiated cells were used as a background control. The irradiation process and parameterization were performed in a spectral range with a specific ratio (UVA/UVB, 95:5%) and an operating temperature between 25 °C and 30 °C, which prevents cell thermal damage. The dose was measured using calibrated sensors (Opsytec) for each channel, which contained a high-precision analogue-to-digital converter, and they were connected to the UV-MAT controller. Since the IC_50_ for the UVA/UVB combined irradiation was defined at 2.04 J/cm^2^ and sufficiently induced phototoxicity in the keratinocytes, an irradiation dose of 2.5 J/cm^2^ (UVA at 2.375 J/cm^2^ and UVB at 0.125 J/cm^2^) was selected for exploring the photoprotective potential of the experimental plant extract and pure compounds. In the current experiment, the irradiation dose was the primary parameter and the exposure time of 30 min corresponds to the point when the sensors detect that the set dose values are reached—for UVA (0:33:20 h/m/s ± 0.15 s) and for UVB (0:13:10 h/m/s ± 0.15 s). For every independent experiment, the time remained the same.

### 4.3. Viability and Photoprotection Assays

To evaluate the maximal safe concentrations of the experimental treatments, the HaCaT cells (1.5 × 10^4^ cells/well) were seeded in 96-well plates and cultured for 48 h to reach confluence. Next, the cells were treated with HRE (1, 5, 10, 20, 50, and 100 µg/mL), CAL, or MYC at 1, 5, 10, 20, 50, and 100 µM or methanol (0.05%, *v*/*v*) as vehicle. On the 24th hour of treatment, an MTT assay was conducted as previously described by Koycheva et al. [56]. In addition, to determine the potential phototoxicity of HRE, CAL, and MYC on the keratinocytes, the viability assay was performed following treatment with the abovementioned concentrations in HaCaT cells exposed to 2.5 J/cm^2^ UVA/UVB radiation for 30 min. All experimental treatments were applied 1 h prior to the UVA/UVB irradiation protocol and remained within the culture media over the next 24 h until viability detection.

### 4.4. Fluorescent Microcopy for UV-Induced ROS Formation

To determine the levels of UVR-induced ROS generation we seeded the HaCaT cells at a density of 3.1 × 10^5^ cells/well in µ-slide 8-well chambers (Ibidi GmbH, Gräfelfing, Germany) with optical bottoms. At the 24th hour of seeding, the cells were pre-treated with MYC or CAL at concentrations of 1, 5, and 10 μM for 1 h and irradiated with 2.5 J/cm^2^ UVR for 30 min. Next, following two PBS washes, 20 μM of the 2′,7′-dichlorofluorescein-diacetate (DCF-DA) solution was added to each well. The dark negative control was included under the same conditions without UV irradiation. Fluorescence was detected after 30 min under a Fluorescent DMi8 inverted microscope from Leica (Wetzlar, Germany) with an FITC filter. Leica LAS X software version 1.4.5 27713 (Wetzlar, Germany) was used for image assessment.

### 4.5. Quantitative Real-Time Polymerase Chain Reaction (RT-qPCR)

Total RNA was isolated for 6 h following UVR exposure using RNAzol RT reagent and reverse transcribed using an NZY First-Strand cDNA Synthesis kit (#MB12502) from NZYTech (Lisbon, Portugal) according to the manufacturer’s instructions. The relative expression of target genes was quantified in fold-change by the comparative threshold cycle (_ΔΔ_CT) method on the CFX96 Touch Real-Time PCR Detection System equipped with CFX Maestro software version 4.1.2433.1219 (Bio-Rad, Hercules, CA, USA) as described previously [41]. Both *GAPDH* and *TUBB* were used as reference genes for normalization. The primer sequences are listed in Supplementary Table S1.

### 4.6. Western Blotting

Total protein lysates were extracted from each group at the 24th hour after UVA/UVB irradiation. The total protein concentration was measured using the Bradford assay. Next, 30 μg per lane of the total protein lysate samples was resolved on SDS-PAGE. Immunoblotting with specific antibodies against STAT1 (#9172) and NRF2 (#12721) from Cell Signalling Technology (Danvers, MA, USA) was performed as described by Koycheva et al. [41]. Normalization was carried out over tubulin as a housekeeping protein (Supplementary Figures S1 and S2). The ChemiDoc MP Imaging System (Bio-Rad, Hercules, CA, USA) was used for multiplex fluorescent detection and Image Lab software 6.0.1 (Bio-Rad) was used for protein quantification.

### 4.7. Statistical Analysis

SigmaPlot software 11.0 (Systat Software GmbH, Erkrath, Germany) was used for data assessment. The results are presented as the mean ± standard error of the mean (SEM). Student’s *t*-test or one-way analysis of variances (ANOVA) with Bonferroni’s post hoc test was utilized for analysis of the differences between groups. Values of * *p* < 0.05 and ** *p* < 0.01 were defined as the significance levels. All experiments were performed in at least three independent biological repeats.

## Data Availability

All relevant data are within the manuscript. The data set generated and analyzed during the current study is also available from the corresponding author upon request.

## Supplementary Materials

The following supporting information can be downloaded at: https://www.mdpi.com/article/10.3390/ijms25042441/s1.
