# Supplementary material for: Myconoside and Calceolarioside E Restrain UV-Induced Skin Photoaging by Activating NRF2-Mediated Defense Mechanisms
Source: Int J Mol Sci. 2024 Feb 19;25(4):2441. doi: 10.3390/ijms25042441 (PMC10888667; doi:10.3390/ijms25042441)
Supplement: Supplementary file 1 [file ijms-25-02441-s001.zip › ijms-2851891-supplementary.pdf]

Myconoside and calceolarioside E protect against UV-induced photoaging by activating NRF2-mediated defense mechanisms in human keratinocytes

Iva D. Stoykova[1,2#], Ivanka K. Koycheva[2#], Biser K. Binev[2], Liliya V. Mihaylova[1,2], Maria Y. Benina[1], Kalina I. Alipieva[3], Milen I. Georgiev[1,2*]

[1] Center of Plant Systems Biology and Biotechnology, 4000 Plovdiv, Bulgaria

[2] Laboratory of Metabolomics, Department of Biotechnology, Institute of Microbiology, Bulgarian Academy of Sciences, 4000 Plovdiv, Bulgaria

[3] Institute of Organic Chemistry with Centre of Phytochemistry, Bulgarian Academy of Sciences, 1113 Sofia, Bulgaria

[#] These authors contributed equally

[*] Corresponding author: Prof. Dr. Milen I. Georgiev, Laboratory of Metabolomics, Department of Biotechnology, Institute of Microbiology, Bulgarian Academy of Sciences, 139 Ruski Blvd, 4000 Plovdiv, Bulgaria; e-mail: milengeorgiev@gbg.bg; tel: 00359 32 64 24 30.

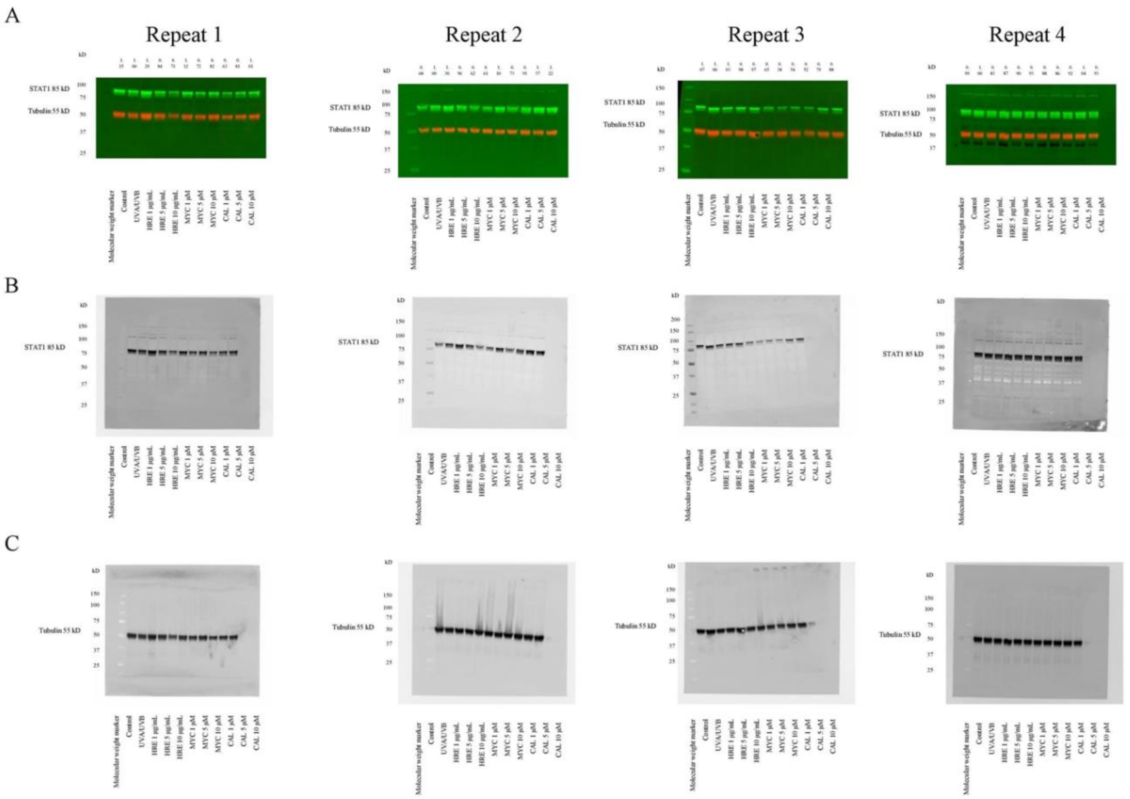

Supplementary Figure S1. Whole uncropped Western blots from which the image for Figure 5A were derived. Four biological repeats for STAT1 protein detection were done from total protein isolated on the 24th hours of UVA/UVB irradiation. In each blot in the first lane the following molecular weight ladder was added: Precision Plus Protein All Blue Standards (#1610373, Bio-Rad). Samples were loaded with 30 µg total protein per lane from left to right as follows: non-irradiated HaCaT human keratinocytes; UVA/UVB-irradiated controls; UVA/UVB stimulated keratinocytes pre-treated with: *Haberlea rhodopensis* Friv. extract (HRE) 1 µg/mL; HRE 5 µg/mL; HRE 10 µg/mL; pure myconoside (MYC) 1 µM; MYC 5 µM; MYC 10 µM; calceolarioside E (CAL) 1 µM; CAL 5 µM and CAL 10 µM. All target proteins were detected with specific anti-STAT1 (#14994) from Cell Signaling Technology (Leiden, The Netherlands) rabbit primary antibodies upon incubation with anti-rabbit secondary fluorescent antibody StarBright 700 (#12004161, Bio-Rad) and are depicted with green color on the multiplex merged images (**A**) and in black on the signle channel blots (**B**). For normalization direct fluorescent detection of tubulin was employed using hFAB™ Rhodamine housekeeping protein fluorescent primary antibodies (#12004164, Bio-Rad) on the same membrane as the target protein depicted in red on the multiplex merged images (**A**) and in black on the signle channel blots (**C**). Processing of the Western blot data was performed with ImageLab 6.0.1 software (Bio-Rad).

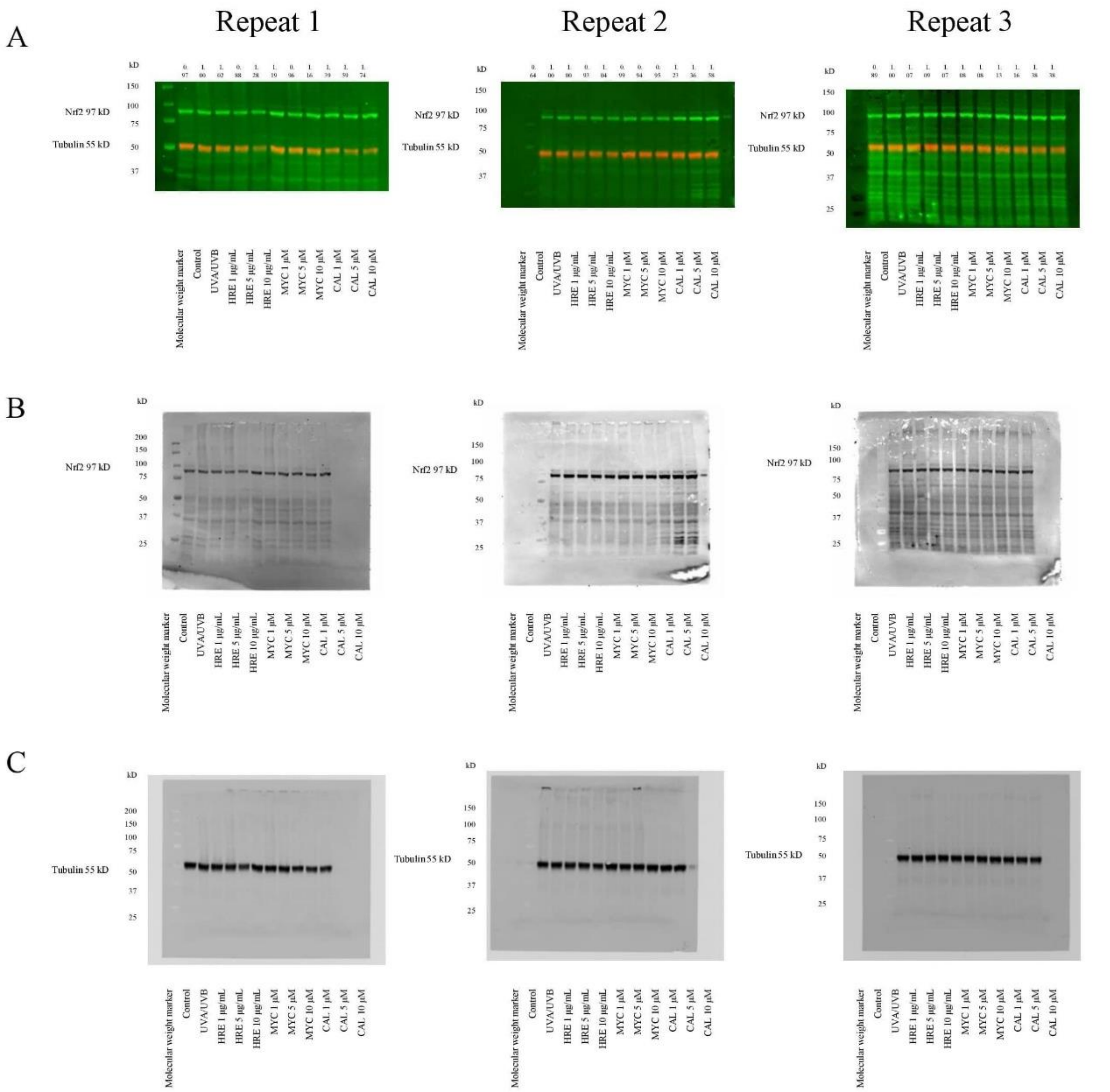

Supplementary Figure S2. Whole uncropped Western blots from which the image for Figure 5B were derived. Three biological repeats for NRF2 protein detection were done from total protein isolated on the 24th hours of UVA/UVB irradiation. In each blot in the first lane the following molecular weight ladder was added: Precision Plus Protein All Blue Standards (#1610373, Bio-Rad). Samples were loaded with 30 µg total protein per lane from left to right as follows: non-irradiated HaCaT human keratinocytes; UVA/UVB-irradiated controls; UVA/UVB stimulated keratinocytes pre-treated with: *Haberlea rhodopensis* Friv. extract (HRE) 1 µg/mL; HRE 5 µg/mL; HRE 10 µg/mL; pure myconoside (MYC) 1 µM; MYC 5 µM; MYC 10 µM; calceolarioside E (CAL) 1 µM; CAL 5 µM and CAL 10 µM. All target proteins were detected with specific anti-NRF2 (#12721) from Cell Signaling Technology (Leiden, The Netherlands) rabbit primary antibodies upon incubation with anti-rabbit secondary fluorescent

antibody StarBright 700 (#12004161, Bio-Rad) and are depicted with green color on the multiplex merged images (**A**) and in black on the signle channel blots (**B**). For normalization direct fluorescent detection of tubulin was employed using hFAB™ Rhodamine housekeeping protein fluorescent primary antibodies (#12004164, Bio-Rad) on the same membrane as the target protein depicted in red on the multiplex merged images (**A**) and in black on the signle channel blots (**C**). Processing of the Western blot data was performed with ImageLab 6.0.1 software (Bio-Rad).

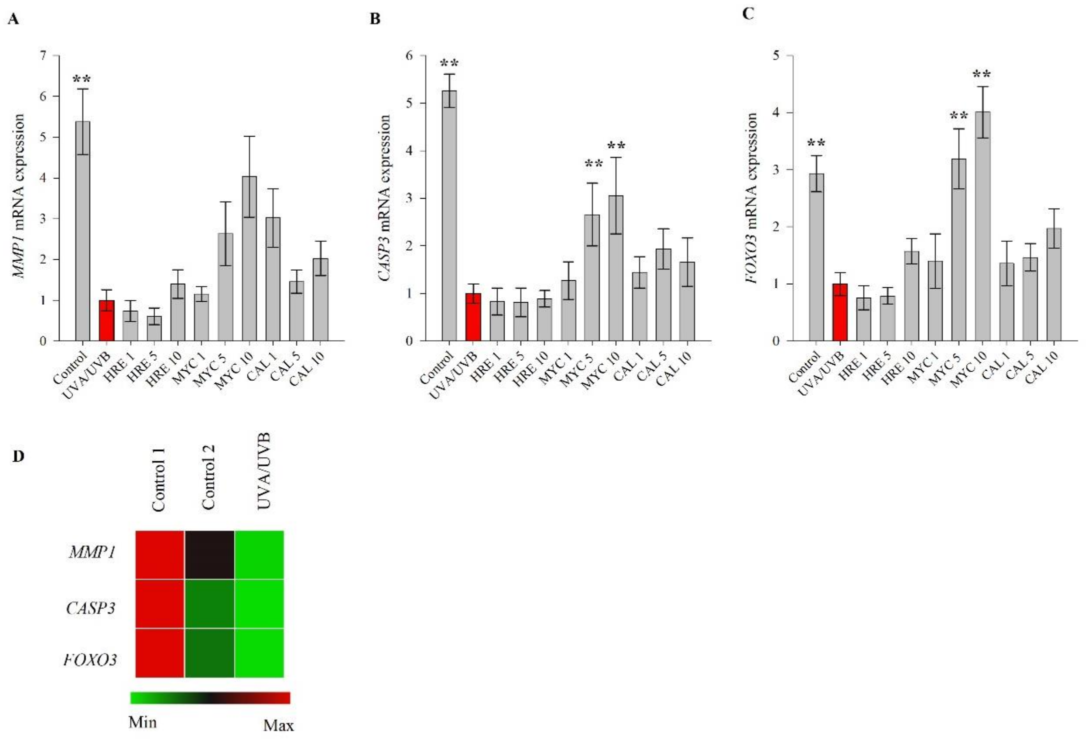

Supplementary Figure S3. Gene expression of *MMP1* (A), *CASP3* (B) and *FOXO3* (C) modulation associated with UVA/UVB-induced photoaging by *H. rhodopensis* extract (HRE), calceolarioside E (CAL) and myconoside (MYC). Control HaCaT cells were plotted over the UVA/UVB-exposed model group to affirm the gene expression profile of the target genes *MMP1*, *CAPS3*, *FOXO3* (**D**). The results are expressed as mean fold change ± SEM compared to UVA/UVB-exposed model group from three independent experiments. **p < 0.01 compared to photoaging model group.

Supplementary Table S1. Primer sequences for the RT-qPCR analysis.

Target gene	Sequence forward primer (5’ - 3’)	Sequence reverse primer (5’ - 3’)
AKT1	CGAGCTGTTCTTCCACCTGT	TAATGTGCCCGTCCTTGTCC
CASP3	GCTCGCAGCTCATACCTGTGGC	CGAAAACCAGAGCGCCGAGTGT

COLA1A	CAAGGTGTTGTGCGATGACG	GTTTCTTGGTCGGTGGGTGA
CTNNB1	GCCCTGGTGAAAATGCTTGG	CGCACTGCCATTTTAGCTCC
FOS	CAGAGCCCCTCACCCTTTCGGA	CACCTTGCCCCTCCTGCCAATG
FOXO1	GACACCAGTTGATCCTGGGG	CTGGCTGCCATAGGTTGACA
FOXO3	ACAAGCTTTTGAGCGCATGG	TTGCCTATGCACGGTTCTGT
GAPDH	CCCACTCCTCCACCTTTGAC	TCCTCTTGTGCTCTTGCTGG
JUN	AGGGGACAAGTCGTCGGAGTCC	CGCAGGCGAACTCCTTCCCAG
JUND	CCCATCGACATGGACACGCAGG	TTCTCTTCCAGGCGCGAGATGC
MMP1	TCTGTTTTCTGGCCACAACTGC	GTCCCTGAACAGCCCAGTACT
MKI67	CGGCTCTCTTTAACTCAGCGCC	GACCAGGTAGGCCAGAGCAAGT
MTOR	CTGCAATCCAGCTGTTTGGCGC	TAGCGCTGCCTTTCGAGATGGC
NFE2L2	TCTGACTCCGGCATTTCACT	GGCACTGTCTAGCTCTTCCA
NQO1	CGACTCCCACAAGGTTGCAG	ACGTCCTCTCTGAGTGAGCC
PGC1A	AAATATCTGACCACAAACGATGACC	GTTGGTTTGGCTTGTAAGTGTTGT
RELA	TTCCAACTGCCCCCAACTTT	TTTGAGTTTCCCCAGCTCCC
SMAD3	TGACTGTGGATGGCTTCACC	CCTCTTCCGATGTGTCTCCG
TGFB1	TACCTGAACCCGTGTTGCTC	GTTGCTGAGGTATCGCCAGG
TIMP1	TGGCATCCTGTTGTTGCTGTGG	AACTTGGCCCTGATGACGAGGT
TUBB	AGCCGTCTTACTCAACTGCC	GTCACCCAGAATGGCAGAA
WNT5A	CGGTGTACAACCTGGCTGAT	GCGCTGTCGTACTTCTCCTT
